# The Representative Points of Generalized Alpha Skew-*t* Distribution and Applications

**DOI:** 10.3390/e26110889

**Published:** 2024-10-22

**Authors:** Yong-Feng Zhou, Yu-Xuan Lin, Kai-Tai Fang, Hong Yin

**Affiliations:** 1 School of Mathematics, Renmin University of China, No. 59, Zhongguancun Street, Haidian District, Beijing 100872, China; zyfeng177@ruc.edu.cn; 2 Research Center for Frontier Fundamental Studies, Zhejiang Lab, Kechuang Avenue, Zhongtai Sub-District, Yuhang District, Hangzhou 311121, China; yuxuanlin@zhejianglab.com; 3 Guangdong Provincial Key Laboratory of Interdisciplinary Research and Application for Data Science, BNU-HKBU United International College, Zhuhai 519087, China; 4 Department of Statistics and Data Science, Faculty of Science and Technology, BNU-HKBU United International College, 2000 Jintong Road, Tangjiawan, Zhuhai 519087, China; ktfang@uic.edu.cn

**Keywords:** entropy, generalized alpha skew-t distribution, kernel density estimation, maximum likelihood estimation, moments, quasi-Monte Carlo, representative points

## Abstract

Assuming the underlying statistical distribution of data is critical in information theory, as it impacts the accuracy and efficiency of communication and the definition of entropy. The real-world data are widely assumed to follow the normal distribution. To better comprehend the skewness of the data, many models more flexible than the normal distribution have been proposed, such as the generalized alpha skew-*t* (GAST) distribution. This paper studies some properties of the GAST distribution, including the calculation of the moments, and the relationship between the number of peaks and the GAST parameters with some proofs. For complex probability distributions, representative points (RPs) are useful due to the convenience of manipulation, computation and analysis. The relative entropy of two probability distributions could have been a good criterion for the purpose of generating RPs of a specific distribution but is not popularly used due to computational complexity. Hence, this paper only provides three ways to obtain RPs of the GAST distribution, Monte Carlo (MC), quasi-Monte Carlo (QMC), and mean square error (MSE). The three types of RPs are utilized in estimating moments and densities of the GAST distribution with known and unknown parameters. The MSE representative points perform the best among all case studies. For unknown parameter cases, a revised maximum likelihood estimation (MLE) method of parameter estimation is compared with the plain MLE method. It indicates that the revised MLE method is suitable for the GAST distribution having a unimodal or unobvious bimodal pattern. This paper includes two real-data applications in which the GAST model appears adaptable to various types of data.

## 1. Introduction

Statistical distributions play a crucial role in information theory since they describe the probability characteristics of data or signals, and hence directly affect the accuracy and efficiency of the representation, transmission, compression, and reconstruction of information. Entropy, as the most important measure in the field of information theory, depends on the statistical distribution of the random variable. In many applications of information theory, it requires the assumption of the statistical distribution of the data. Although assumed to follow the normal distribution in most statistical analyses due to mathematical convenience and generality, real-world data frequently exhibit skewness, leading to the demand for more flexible models. The geometric Brownian motion (GBM) as a popular model of stochastic processes assumes that its solutions follow the log-normal distribution. Gupta et al. (2024) [1] indicated that the GBM yields trajectories significantly deviated from the reference distribution when the data do not meet the log-normal assumption. To deal with the limitations in such a scenario, some may consider correcting the model as in [1]. Constructing alternative distributions of the normal distribution has been a common concern.

The skew-normal (SN) distribution is an extension of the normal distribution that allows for skewness, capable of modeling asymmetric data. It was first introduced by Azzalini (1985) [2]. If a random variable *Z* has a probability density function (pdf) given by

(1) ϕ(z;s)=2ϕ(z)Φ(sz),z∈R,s∈R,

where ϕ(·) and Φ(·) are the pdf and cumulative distribution function (cdf) of the standard normal distribution, then *Z* follows the SN distribution, denoted as Z∼SN(s). The parameter *s* controls the skewness of the distribution. When s=0, the SN distribution reduces to the standard normal distribution. With s>0, the SN distribution is right-skewed, while s<0 implies left skewness.

The skew-*t* (ST) distribution is an intriguing example among scale mixtures of SN distributions. It was first formulated by Branco and Dey (2001) [3] and later extensively studied by Azzalini and Capitanio (2003) [4]. An ST random variable, Y∼ST(s,ν), can be represented as

(2) $$Y=\frac{Z}{\sqrt{V/\nu }},$$

where Z∼SN(s) and $V∼\chi_{\nu }^{2}$, i.e., chi-square distribution with ν degrees of freedom, are independent of each other. The moment of *Y* exists only when the order is less than ν, which is the same condition required as the Student’s *t*-distribution with ν degrees of freedom, denoted by $t_{\nu }$. The construction method from the SN distribution to the ST distribution is similar to the approach used to derive the Student’s *t*-distribution from the normal distribution. The pdf of the ST distribution is given by

(3) $$f\left(y;s,\nu \right)=2t\left(y;\nu \right)T\left(\sqrt{\frac{1+\nu }{y^{2}+\nu }}sy;\nu +1\right),\;y\in R,\;s\in R,$$

where t(·) is the pdf of $t_{\nu }$, and T(·) is the cdf of $t_{\nu +1}$. The parameter ν controls the tail heaviness. As ν approaches infinity, the ST distribution approaches the SN distribution. Lower values of ν result in heavier tails, providing robustness against outliers. Similar to the SN distribution, the parameter *s* controls the skewness. When s=0, the ST distribution reduces to the Student’s *t*-distribution. Azzalini and Genton (2008) [5] conducted a quite extensive numerical exploration, demonstrating that the ST distribution can adapt well to various empirical problems. They utilized an autoregressive model of order one, $Y\left(t\right)=\beta_{0}+\beta_{1}Y\left(t-1\right)+\epsilon \left(t\right)$ with $\beta_{0}\in R$ and $|\beta_{1}|\leq 1$, to fit the 91 monthly interest rates of an Austrian bank. Their results clearly showed that the error components ϵ(t) have an ST distribution, where the small degrees of freedom parameter signifies heavy tails in the error distribution, allowing the ST model to better manage outliers than the normal distribution. The ST distribution, which combines the characteristics of the Student’s *t*-distribution and the SN distribution, is particularly suitable for the applications in finance that need to model returns with skewness and excess kurtosis, as well as in environmental studies where the focus is on modeling extreme events. Martínez-Flórez et al. (2020) [6] also mentioned other kinds of skew distributions like skew-Student-*t* distribution, skew-Cauchy distribution, skew-logistic distribution and skew-Laplace distribution. They summarized those distributions as skew-elliptical distributions since those distributions have a unified expression form of the density function as

$$h_{Y}\left(y;s\right)=2f\left(y\right)F\left(sy\right),\;y,s\in R,$$

where f(·) is a symmetric pdf, and F(·) is the corresponding cdf.

Another type of skew distribution is to add a coefficient function with an α argument to the density function. Elal-Olivero (2010) [7] proposed a distribution called alpha-skew-normal (ASN), with a pdf defined as

(4) $$f\left(x;\alpha \right)=\frac{{\left(1-\alpha x\right)}^{2}+1}{2+\alpha^{2}}\phi \left(x\right),\;x\in R,\;\alpha \in R.$$

If a random variable *X* has the pdf as (4), we denote it as X∼ASN(α). This distribution is more flexible than SN and ST distributions since it can be unimodal or multimodal by adjusting the α parameter. When α=0, the ASN distribution reduces to the standard normal distribution, X∼N(0,1).

Although the ASN distribution is able to model both skew and bimodal data, it has limitations when data have tails thinner or thicker than the normal distribution. In order to fit stock data more accurately, Altun et al. (2018) [8] introduced a new generalized alpha skew-*t* (GAST) distribution combining the approaches of [4,7]. They combined the GAST distribution with the generalized autoregressive conditional heteroskedasticity (GARCH) model to build a new Value-at-Risk (VaR) prediction model for forecasting daily log returns in three years. They compared the failure rates of the GARCH models under different distribution assumptions including normal, Student’s *t*, ST and GAST. The results showed that the GAST distribution performs the best in the backtesting. The definition of GAST distribution and its properties with proof will be elaborated in the next section.

For an unknown continuous statistical distribution, an empirical distribution of a random sample is a traditional way to approximate the target distribution. However, it often leads to low accuracy, and hence the support points for the discrete approximation, also known as representative points (RPs), are explored in order to preserve the information of the target distribution as much as possible. Representative points have a big potential for applications in statistical simulation and inference, see Fang and Pan (2023) [9] for a comprehensive review. Various kinds of representative points of different statistical distributions have been explored in the literature. Especially for complex distributions, the study on the representative points is necessary. The concept of representative points is to simplify complex probability distributions with discrete points easier to manipulate, facilitating efficient computations and analyses. These points serve as a finite set that approximates the distribution of a random variable that can be either discrete or continuous and either univariate or multivariate. In this paper, we focus on the study of the representative points of the GAST distribution and applications. We first introduce the concepts of three kinds of RPs here, while the specific construction procedures are included in Section 4 with their applications on the estimation of moments and densities.

There are many existing criteria for choosing RPs of a distribution, such as Monte Carlo RPs (MC-RPs), quasi-Monte Carlo RPs (QMC-RPs) and mean square error RPs (MSE-RPs) that will be introduced as follows. In fact, the Kullback–Leibler (KL) divergence or relative entropy of two probability distributions is a good criterion for this purpose. The entropy has been utilized as a measure of the experimental design, for example, Lin et al. (2022) [10]. Due to computational complexity, entropy is not popularly used in generating RPs in applications. Therefore, in this article, we study MC-RPs, QMC-RPs, and MSE-RPs of the Generalized alpha skew-*t* distribution only.

### 1.1. Monte Carlo Representative Points

Let *X* be the population random variable with the cdf F(x)=P{X≤x},x∈R. Various Monte Carlo methods provide ways to generate independent identically distributed (i.i.d.) samples {$x_{1},\ldots ,x_{n}$} from the population, and $p\left(x_{i}\right)=\frac{1}{n}$, i=1,…,n. The empirical distribution of the random sample is defined as follows:

$$F_{n}\left(x\right)=\frac{1}{n}\sum_{i=1}^{n}I_{\left\{x_{i}\leq x\right\}},$$

where $I_{A}$ is the indicator function of *A*. The empirical distribution $F_{n}\left(x\right)$ should be close to F(x) in the sense of consistency. Hence, $F_{n}\left(x\right)$ can be regarded as an approximation of F(x). We denote this empirical distribution of random samples generated by the Monte Carlo method as $F_{MC}$. Traditional statistical inference is based on the empirical distribution. Efron (1979) [11] proposed a resampling technique, *the bootstrap* method, with which we can take a set of random samples from $F_{MC}$ instead of *F*. Combined with bootstrap, the MC-RPs have proven to be useful in statistical inference, such as parameter estimation, density estimation and hypothesis testing. However, the MC method has many limitations since the convergence rate of $F_{n}\left(x\right)\to F\left(x\right)$ in distribution as n→∞, given by $O(\frac{1}{\sqrt{n}})$, is too slow. The following two kinds of RPs improve the convergence rate nicely.

### 1.2. Quasi-Monte Carlo Representative Points

For a high-dimensional integration problem:

$$I\left(f\right)=\int_{0}^{1}\cdots \int_{0}^{1}f\left(y_{1},\ldots ,y_{d}\right)dy_{1}\cdots dy_{d}=\int_{C^{d}}f\left(y\right)dy,$$

where *f* is a continuous function on $C^{d}={\left[0,1\right]}^{d}$. Suppose that $Y=\{y_{1},\ldots ,y_{n}\}$ is a set of *n* points uniformly scattered in $C^{d}$, we can estimate I(f) by

$$\bar{f(y)}=\frac{1}{n}\sum_{i=1}^{n}f\left(y_{i}\right),\;y_{i}\in Y.$$

If we generate Y by the MC method, the convergence rate of $\bar{f(y)}\to I\left(f\right)$ is $O(1/\sqrt{n})$ as n→∞. The quasi-Monte Carlo (QMC) method provides many ways for the construction of Y to increase the convergence rate. Through the QMC method, the convergence rate can reach $O(n^{-1}\log^{d}n)$ according to Fang et al. (1994) [12]. For further theory studies, readers can refer to Hua and Wang (1981) [13] and Niederreiter (1992) [14]. In the study of [12], the *F*-discrepancy is used to measure the uniformity of Y in $C^{d}$, which is defined by

(5) $$D\left(F,F_{n}\right)=\underset{x\in R^{d}}{\sup}\left|F\left(x\right)-F_{n}\left(x\right)\right|,$$

where F(x) is the cdf of uniform distribution $U(C^{d})$ and $F_{n}\left(x\right)$ is the empirical distribution of Y. The Y that minimizes $D(F,F_{n})$ is called QMC-RPs which have equal probability 1/n.

For the univariate distribution of this paper, the QMC method is designed to sample points that are uniformly distributed on the interval [0,1]. If the inverse function of *F* exists, then the set of *n* points:

(6) $$\left\{b_{j}=F^{-1}\left(\frac{2j-1}{2n}\right),j=1,\ldots ,n\right\},$$

has been proved to have the minimal *F*-discrepancy of 1/2n from F(x) [12]. Therefore, the set of points $B=\{b_{1},\cdots ,b_{n}\}$ is called the QMC-RPs of F(x). Fang et al. (1994) [12] gave a comprehensive study on QMC methods and their applications in statistical inference, experimental design, geometric probability, and optimization.

### 1.3. Mean Square Error Representative Points

The concept of MSE-RPs was independently proposed by Cox (1957) [15], Flury (1990) [16] and many others. In the literature, “MSE-RPs” have been called by different names, such as “quantized” and “principal points”. Let a random variable X∼F(x) with finite mean μ and variance $\sigma^{2}$. To provide the best representation of *F* for a given number *n*, we select a set of *n* representative points having the least mean square error from F(x), and form a discrete distribution $F_{MSE}^{\left(n\right)}$. Denote $Y_{MSE}^{\left(n\right)}∼F_{MSE}^{\left(n\right)}$ defined as

$$F_{MSE}^{\left(n\right)}\left(y\right)=\sum_{i=1}^{n}p_{i}^{\left(n\right)}I_{\left\{b_{i}^{\left(n\right)}\leq y\right\}},$$

with the probability mass function

(7) $$f\left(y=b_{i}^{\left(n\right)}\right)=p_{i}^{\left(n\right)},\;i=1,\cdots ,n,$$

where $-\infty <b_{1}^{\left(n\right)}<b_{2}^{\left(n\right)}<\cdots <b_{n}^{\left(n\right)}<\infty$ are MSE-RPs of *X* and $p_{1}^{\left(n\right)},\cdots ,p_{n}^{\left(n\right)}$ are the corresponding probabilities with respect to

(8) $$MSE\left(Y_{MSE}\right)=MSE\left(b_{1}^{\left(n\right)},\cdots ,b_{n}^{\left(n\right)}\right)=\int_{-\infty }^{\infty }\min_{i=1,\cdots ,n}{\left(x-b_{i}^{\left(n\right)}\right)}^{2}f\left(x\right)dx,$$

and

$$\begin{array}{cc} \; & p_{1}^{\left(n\right)}=\int_{-\infty }^{(b_{1}^{\left(n\right)}+b_{2}^{\left(n\right)})/2}f\left(x\right)dx,\\ \; & p_{i}^{\left(n\right)}=\int_{(b_{i-1}^{\left(n\right)}+b_{i}^{\left(n\right)})/2}^{(b_{i}^{\left(n\right)}+b_{i+1}^{\left(n\right)})/2}f\left(x\right)dx,\;i=2,\ldots ,n-1,\\ \; & p_{n}^{\left(n\right)}=\int_{(b_{n-1}^{\left(n\right)}+b_{n}^{\left(n\right)})/2}^{+\infty }f\left(x\right)dx. \end{array}$$

The MSE-RPs have many useful properties. Graf and Luschgy (2007) [17], and Fei (1991) [18] proved that

(9) $$E\left[Y_{MSE}^{\left(n\right)}\right]=E\left[X\right],\;\lim_{n\to \infty }E\left[{\left(X-Y_{MSE}^{\left(n\right)}\right)}^{2}\right]=\lim_{n\to \infty }\left[\mathrm{Var}\left(X\right)-\mathrm{Var}\left(Y_{MSE}^{\left(n\right)}\right)\right]=0.$$

Hence, $Y_{MSE}^{\left(n\right)}$ converges to *X* in distribution.

In this paper, Section 2 begins by reviewing the definition and properties of the GAST distribution. To explore the relationship between the classification of the GAST distribution and the three parameters α,s,ν, we apply the uniform design (Wang and Fang 1981 [19]) to arrange the values of parameter combinations, and then depict the corresponding density plots. Section 2 also classifies the GAST distribution according to the number of peaks in the density function with some proofs. The first four order moments and stochastic representation of the GAST distribution are shown in this section. Section 3 mainly introduces a maximum likelihood estimation (MLE) method with a distribution-free quantile estimator: QMC-MLE (Li and Fang 2024 [20]). In this QMC-MLE method, the estimated quantiles of the sample are used to replace the original sample, and then the MLE is performed on the estimated quantiles to obtain the parameter estimates. We explore the parameter estimation effectiveness of QMC-MLE for small samples by simulation in this section. In order to cover both unimodal and bimodal cases, we choose the GAST distribution with different parameter settings as the underlying distributions. In this section, we find that the effectiveness of QMC-MLE in parameter estimation is influenced by the number of peaks of sample. Section 4 calculates the three types of RPs, MC-RPs, QMC-RPs, and MSE-RPs, of the GAST distribution for different sample size *n*. For MSE-RPs, the calculation process requires a parametric k-means algorithm (Stampfer and Stadlober 2002 [21]). We will compare the estimates of four statistics (mean, variance, skewness and kurtosis) by the three types of RPs of the underlying distributions. Another application of RPs is density estimation. Section 4 combines the kernel density method (Rosenblatt 1956 [22]) and the three types of RPs to estimate the density of the underlying GAST distributions. Section 5 applies the RPs to real data samples to show the outstanding performance of MSE-RPs under the assumption of a GAST model.

## 2. Generalized Alpha Skew-t Distribution

In this section, we give the definition of the density function of the GAST distribution (Altun et al., 2018 [8]) and list some of its commonly used subdistributions. We set the parameter values by the uniform design method (Wang and Fang 1981 [19]) to fully demonstrate the influence of parameters on the shape of the density function. Section 2.2 discusses how the parameters influence the number of peaks of density under four conditions. Section 2.3 and Section 2.4 give the moments and stochastic representation of the GAST distribution, respectively.

### 2.1. Definition of the GAST Distribution

**Definition** **1.** *(GAST distribution). A random variable X is said to follow the GAST distribution, denoted as X∼GAST(α,s,ν), if it has the following pdf*

(10) $$f\left(x;\alpha ,s,\nu \right)=\frac{{\left(1-\alpha x\right)}^{2}+1}{c(\alpha ,s,\nu )}t\left(x;\nu \right)T\left(\sqrt{\frac{1+\nu }{x^{2}+\nu }}sx;\nu +1\right),\;\nu >2,\;x\in R,$$

*where*

(11) $$c\left(\alpha ,s,\nu \right)=1-\alpha \left[\frac{s}{\sqrt{1+s^{2}}}{\left(\frac{\nu }{\pi }\right)}^{1/2}\Gamma \left(\frac{\nu -1}{2}\right)/\Gamma \left(\frac{\nu }{2}\right)\right]+\frac{\alpha^{2}}{2}\left(\frac{\nu }{\nu -2}\right).$$

**Proposition** **1.** *If a random variable Y∼ST(s,ν), the c(α,s,ν) in* (11) *can be written as*

(12) $$c\left(\alpha ,s,\nu \right)=1-\alpha E\left[Y\right]+\frac{\alpha^{2}}{2}E\left[Y^{2}\right].$$

**Proof.**  We set a random variable Z∼SN(α). From the Equation (2), the moments of the ST distribution are given by

(13) $$E\left[Y^{m}\right]=\frac{{\left(\nu /2\right)}^{m/2}\Gamma \left(\frac{\nu -m}{2}\right)}{\Gamma (\frac{\nu }{2})}E\left[Z^{m}\right].$$

Henze (1986) [23] has given the general expression of the odd moments of *Z*, which is

(14) $$E\left[Z^{2k+1}\right]=\sqrt{\frac{2}{\pi }}\frac{\delta }{{\left(1+s^{2}\right)}^{k}}\frac{(2k+1)!}{2^{k}}\sum_{i=0}^{k}\frac{i!{\left(2s\right)}^{2i}}{(2i+1)!(k-i)!},\;k=0,1,\cdots ,n,$$

where $\delta =s/\sqrt{1+s^{2}}$. The even moments coincide with the standard normal distribution, because $Z^{2}∼\chi_{1}^{2}$. Hence, the first two moments of ST distribution are, respectively, given by

$$\begin{array}{cc} \; & E\left[Y\right]=\frac{{\left(\frac{\nu }{\pi }\right)}^{1/2}\Gamma \left(\frac{\nu -1}{2}\right)}{\Gamma \left(\frac{\nu }{2}\right)}\delta ,\\ \; & E\left[Y^{2}\right]=\frac{\nu }{\nu -2}. \end{array}$$

Then the Equation (12) is proved.    □

The GAST distribution involves several popular useful distributions:

- If α=0, the GAST distribution reduces to the skew-*t* (ST) distribution.
- If s=0, the GAST distribution reduces to the alpha-skew-*t* (AST) distribution.
- If α=0 and s=0, the GAST distribution reduces to the Student’s-*t* distribution.
- If ν→∞, the GAST distribution reduces to the alpha-skew-normal (ASN) distribution.
- If ν→∞, α=0, the GAST distribution reduces to the skew-normal (SN) distribution.
- If ν→∞, α=0 and s=0 the GAST distribution reduces to the normal distribution.

In order to depict the GAST densities, especially the characteristics of unimodal or multimodal with different combinations of parameters, the experimental design is used to arrange the parameter values. The uniform design is a number-theoretic method, proposed by Wang and Fang (1981) [19]. As a robust experimental design method, the uniform design has been widely applied in various fields. A uniform design table provides a scientific arrangement of experiments by tabulating the level combinations of factors of interest. Let $U_{n}\left(q^{s}\right)$ denote a uniform design with *n* experimental runs and *s* factors each having *q* levels. The uniform design table, $U_{16}\left(16^{3}\right)$, adopted in this paper is derived from the website Uniform-Design-Tables (https://fst.uic.edu.cn/isci/research/Uniform_Design_Tables.htm (accessed on 15 September 2024)). In uniform design tables, the levels of factors are labeled by positive integers. For a unit hypercube experimental region ${\left[0,1\right]}^{s}$, the levels {1,2,…,q} usually take values $\left\{\frac{1}{2q},\frac{3}{2q},\ldots ,\frac{2q-1}{2q}\right\}$. For any hyperrectangle experimental region ${\left[a,b\right]}^{s}$, a linear transformation $a+\left(b-a\right)\frac{i}{2q},\;i=1,3,\ldots ,2q-1$ is applied. Table 1 lists the arrangement of the uniform design table $U_{16}\left(16^{3}\right)$ for the parametric region, α×s×ν:[−2.6,3.8]×[−3,3.4]×[2.5,18.5], indicating the 16 kinds of parameter settings.

**Figure 1:**
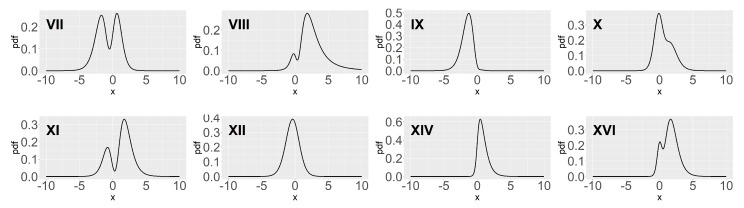
Some plots of GAST densities with parameters in Table 1.

Figure 1 shows the density plots corresponding to eight parameter settings in Table 1, which are enough to represent the plot of GAST density. As shown in Figure 1, there are four cases in which the pdfs are bimodal and the No. XII and XIV GAST distributions are AST and ST distributions, respectively.

In Section 2.2, we will show how the parameters α,s and ν affect the number of peaks of two special types of the GAST distribution, the AST and ST distributions, leading to the two categories, unimodal and bimodal. The number of peaks in the distribution may affect the parameter estimation. For instance, if the sample size is small and the density function presents a bimodal shape, then the sample is likely to miss the turning points, which will affect the parameter estimation to a certain extent. In addition, the calculation of representative points will also be affected, and the accuracy of derivative density estimation may be reduced.

### 2.2. Unimodal and Bimodal Properties

Since the density plots of the GAST distribution are varied, we will divide the GAST distribution into two categories: unimodal and bimodal. The number of peaks is determined by the number of zeros of the first derivative of (10). If it has one zero, then the density function is unimodal. If it has three zeros, then the density function is bimodal. To simplify the analysis, we consider the situations under four different parameter combinations of α and *s*, (α=0,s=0), (α=0,s≠0), (α≠0,s=0) and (α≠0,s≠0). The discussion is as follows:

(1) α=0,s=0 and X∼t(ν)

The Student’s *t*-distribution is a well-known unimodal distribution.

(2) α=0,s≠0 and X∼ST(s,ν)

The pdf of X∼ST(s,ν), f(x;s,ν), is given by (3), ν>2.

**Proposition** **2.** *The skew-t distribution is always unimodal.*

**Proof.**  We derive the first derivative of (3) as follows:

(15) $$f^{\prime }\left(x;s,\nu \right)=2t^{\prime }\left(x;\nu \right)T\left(sx\sqrt{\frac{\nu +1}{\nu +x^{2}}};\nu +1\right)+2t\left(x;\nu \right)T^{\prime }\left(sx\sqrt{\frac{\nu +1}{\nu +x^{2}}};\nu +1\right),$$

where

(16) $$\begin{array}{cc} t(x;\nu ) & =\frac{\Gamma \left(\frac{\nu +1}{2}\right)}{\sqrt{\pi \nu }\Gamma (\frac{\nu }{2})}{\left(1+\frac{x^{2}}{\nu }\right)}^{-\frac{\nu +1}{2}}\equiv c{\left(1+\frac{x^{2}}{\nu }\right)}^{-\frac{\nu +1}{2}}, \end{array}$$

(17) $$\begin{array}{cc} t^{\prime }\left(x;\nu \right) & =-c\left(\frac{2x}{\nu }\right)\left(\frac{\nu +1}{2}\right){\left(1+\frac{x^{2}}{\nu }\right)}^{-\frac{\nu +1}{2}-1}=t\left(x;\nu \right)\left(-x\right)\left(\frac{\nu +1}{\nu +x^{2}}\right), \end{array}$$

(18) $$\begin{array}{cc} T^{\prime }\left(\cdot \right) & =\left(s\sqrt{\frac{\nu +1}{\nu +x^{2}}}\right)\left(\frac{\nu }{\nu +x^{2}}\right)t\left(sx\sqrt{\frac{\nu +1}{\nu +x^{2}}};\nu +1\right). \end{array}$$

Substituting (17) and (18) in Equation (15), we obtain that

$$\begin{array}{cc} f^{\prime }\left(x;s,\nu \right) & =2\left(-x\right)t\left(x;\nu \right)\left(\frac{\nu +1}{\nu }\right)\left(\frac{\nu }{\nu +x^{2}}\right)T\left(\cdot \right)\\ \; & \;+2t\left(x;\nu \right)\left(s\sqrt{\frac{\nu +1}{\nu +x^{2}}}\right)\left(\frac{\nu }{\nu +x^{2}}\right)t\left(sx\sqrt{\frac{\nu +1}{\nu +x^{2}}};\nu +1\right)\\ \; & =2t\left(x;\nu \right)\left(\frac{\nu }{\nu +x^{2}}\right)\left[\left(-x\right)\left(\frac{\nu +1}{\nu }\right)T\left(\cdot \right)+\left(s\sqrt{\frac{\nu +1}{\nu +x^{2}}}\right)t\left(sx\sqrt{\frac{\nu +1}{\nu +x^{2}}};\nu +1\right)\right]. \end{array}$$

As $2t\left(x;\nu \right)\left(\frac{\nu }{\nu +x^{2}}\right)>0$, the solution to $f^{\prime }\left(x;s,\nu \right)=0$ can be evaluated by solving the next equation:

(19) $$g\left(x\right)=\left(-x\right)\left(\frac{\nu +1}{\nu }\right)T\left(\cdot \right)+\left(s\sqrt{\frac{\nu +1}{\nu +x^{2}}}\right)t\left(sx\sqrt{\frac{\nu +1}{\nu +x^{2}}};\nu +1\right)=0.$$

Since f(x;s,ν) is symmetric with respect to *s*:

f(x;s,ν)=f(−x;−s,ν).

The number of peaks is not affected by the sign of *s* such that we assume s>0. From the expression of (19), we can see that g(x)>0, when x∈(−∞,0]. When x∈(0,+∞), we have

$${\left(sx\sqrt{\frac{\nu +1}{\nu +x^{2}}}\right)}^{\prime }=\left(s\sqrt{\frac{\nu +1}{\nu +x^{2}}}\right)\left(\frac{\nu }{\nu +x^{2}}\right)>0.$$

Hence, T(·) is monotonically increasing, while t(·) is monotonically decreasing. Therefore, we can deduce that g(x) is decreasing when x∈(0,+∞), i.e., g(x)→−∞, as x→+∞. Hence, there is only one solution $x_{1}$, s.t. $g(x_{1})=0$. And the ST distribution must be unimodal.    □

(3) α≠0,s=0 and X∼AST(α,ν)

The pdf of X∼AST(α,ν) is given by

(20) $$f\left(x;\alpha ,\nu \right)=\frac{{\left(1-\alpha x\right)}^{2}+1}{2c(\alpha ,0,\nu )}t\left(x;\nu \right)\;,\nu >2.$$

**Proposition** **3.** *The pdf of the AST distribution, f(x;α,ν) as (20), is bimodal if and only if $g\left(x_{1}\right)<0<g\left(x_{2}\right)$ and $\alpha \notin \left(-\sqrt{\frac{\nu^{2}-3}{3\nu^{2}-3\nu }},\sqrt{\frac{\nu^{2}-3}{3\nu^{2}-3\nu }}\right)$, where*

(21) $$\begin{array}{cc} g(x) & =\left(1-\nu \right)\alpha^{2}x^{3}+2\alpha \nu x^{2}+\left(2\alpha^{2}\nu -2\nu -2\right)x-2\alpha \nu ,\\ x_{1},x_{2} & =\frac{-4\alpha \nu \mp \sqrt{\Delta }}{2\alpha^{2}\left(3-3\nu \right)},\;\Delta =8\alpha^{2}\left[\left(3\nu^{2}-3\nu \right)\alpha^{2}+\left(3-\nu^{2}\right)\right]. \end{array}$$

*Otherwise, it is unimodal. It is worth mentioning that a sufficient condition for f(x;α,ν) to be unimodal is*

(22) $$\alpha \in \left(-\sqrt{\frac{\nu^{2}-3}{3\nu^{2}-3\nu }},\sqrt{\frac{\nu^{2}-3}{3\nu^{2}-3\nu }}\right).$$

**Proof.**  Differentiating (20), we obtain

$$f^{\prime }\left(x;\alpha ,\nu \right)=\frac{1}{2c(\alpha ,0,\nu )}\left[\left(-2\alpha \right)\left(1-\alpha x\right)t\left(x;\nu \right)-\frac{(\nu +1)x}{\nu +x^{2}}t\left(x;\nu \right)\left({\left(1-\alpha x\right)}^{2}+1\right)\right].$$

Since $\frac{1}{2c(\alpha ,0,\nu )}$ is a constant, and t(x;ν)>0, we obtain the equivalent relation expression

(23) $$\begin{array}{cc} \; & f^{\prime }\left(x;\alpha ,\nu \right)=0\Leftrightarrow \left(-2\alpha \right)\left(1-\alpha x\right)\left(\nu +x^{2}\right)-\left(\nu +1\right)x\left({\left(1-\alpha x\right)}^{2}+1\right)=0\\ \; & \Leftrightarrow g\left(x\right)\equiv \left(1-\nu \right)\alpha^{2}x^{3}+2\alpha \nu x^{2}+\left(2\alpha^{2}\nu -2\nu -2\right)x-2\alpha \nu =0. \end{array}$$

Now our problem is transformed into studying the number of zeros of the function g(x). The first derivative of (23) is

$$g^{\prime }\left(x\right)=\left(3-3\nu \right)\alpha^{2}x^{2}+4\alpha \nu x+2\alpha^{2}\nu -2\nu -2\;,\;\nu >2.$$

This is a quadratic function with a downward opening. The discriminant Δ of $g^{\prime }\left(x\right)$ is as follows:

$$\Delta =8\alpha^{2}\left[\left(3\nu^{2}-3\nu \right)\alpha^{2}+\left(3-\nu^{2}\right)\right].$$

If Δ<0, namely

$$-\sqrt{\frac{\nu^{2}-3}{3\nu^{2}-3\nu }}<\alpha <\sqrt{\frac{\nu^{2}-3}{3\nu^{2}-3\nu }},$$

then g(x) is monotonically decreasing. Since $\lim_{x\to -\infty }g\left(x\right)=+\infty$, $\lim_{x\to +\infty }g\left(x\right)=-\infty$, there must be only one root of g(x)=0, i.e., f(x;α,ν) is unimodal. It is worth mentioning that the parameter setting of No.XII in Table 1 fits this condition.

If Δ>0, then

$$\exists \;x_{1}<x_{2},\;s.t.\;g^{\prime }\left(x_{1}\right)=g^{\prime }\left(x_{2}\right)=0,\;\mathrm{where}\;x_{1},x_{2}=\frac{-4\alpha \nu \mp \sqrt{\Delta }}{2\alpha^{2}\left(3-3\nu \right)}.$$

We can obtain that

$$\begin{array}{cc} \; & g^{\prime }\left(x\right)>0,\;x\in \left(x_{1},x_{2}\right),\\ \; & g^{\prime }\left(x\right)<0,\;x\in \left(-\infty ,x_{1}\right)\cup \left(x_{2},\infty \right). \end{array}$$

Hence, when $x\in (x_{1},x_{2})$, g(x) is monotonically increasing. When $x\in \left(-\infty ,x_{1}\right)\cup \left(x_{2},\infty \right)$, g(x) is monotonically decreasing. If $g\left(x_{1}\right)<0<g\left(x_{2}\right)$, then g(x) has three zeros, and f(x;α,ν) is bimodal. If condition (22) is met, f(x;α,ν) is unimodal. To sum up, f(x;α,ν) is bimodal if and only if Δ>0, i.e., condition (22) is not satisfied, and $g\left(x_{1}\right)<0<g\left(x_{2}\right)$. Otherwise, it is unimodal.    □

(4) α≠0,s≠0 and X∼GAST(α,s,ν)

Differentiating the pdf of the GAST distribution as (10), we obtain

$$\begin{array}{cc} f^{\prime }\left(x;\alpha ,s,\nu \right)=\frac{t(x;\nu )}{c(\alpha ,s,\nu )}\{\; & \left(-2\alpha \right)\left(1-\alpha x\right)T\left(\cdot \right)+\left(\frac{-(\nu +1)x}{\nu +x^{2}}\right)\left({\left(1-\alpha x\right)}^{2}+1\right)T\left(\cdot \right)\\ \; & +\left({\left(1-\alpha x\right)}^{2}+1\right)\left(s\sqrt{\frac{\nu +1}{\nu +x^{2}}}\right)\left(\frac{\nu }{\nu +x^{2}}\right)t\left(sx\sqrt{\frac{\nu +1}{\nu +x^{2}}};\nu +1\right)\}. \end{array}$$

Let

(24) $$\begin{array}{cc} g(x)= & \left(-2\alpha \right)\left(1-\alpha x\right)T\left(\cdot \right)+\left(\frac{-(\nu +1)x}{\nu +x^{2}}\right)\left({\left(1-\alpha x\right)}^{2}+1\right)T\left(\cdot \right)+\\ \; & \left({\left(1-\alpha x\right)}^{2}+1\right)\left(s\sqrt{\frac{\nu +1}{\nu +x^{2}}}\right)\left(\frac{\nu }{\nu +x^{2}}\right)t\left(sx\sqrt{\frac{\nu +1}{\nu +x^{2}}};\nu +1\right). \end{array}$$

Then we have $f^{\prime }\left(x;\alpha ,s,\nu \right)=\frac{t(x;\nu )}{c(\alpha ,s,\nu )}g\left(x\right)$, and we obtain

$$f^{\prime }\left(x;\alpha ,s,\nu \right)=0\Leftrightarrow g\left(x\right)=0.$$

Due to the complexity of the g(x), it is difficult to study its zeros. The discussion of such a situation remains to be studied.

### 2.3. Moments of the GAST Distribution

From the pdf of the GAST distribution in (10), the *k*th moment of X∼GAST(α,s,ν) is given by

(25) $$E\left[X^{k}\right]=\frac{E\left[Y^{k}\right]-\alpha E\left[Y^{k+1}\right]+\frac{\alpha^{2}}{2}E\left[Y^{k+2}\right]}{c(\alpha ,s,\nu )}\equiv \frac{M_{k}\left(\alpha ,s,\nu \right)}{c(\alpha ,s,\nu )},$$

where Y∼ST(s,ν). Combined with (13) and (14), we have the first four moments of *X* in which the $M_{k}\left(\alpha ,s,\nu \right)$ is given as follows  

$$\begin{array}{cc} M_{1}\left(\alpha ,s,\nu \right)= & \delta {\left(\frac{\nu }{\pi }\right)}^{1/2}\frac{\Gamma \left(\frac{\nu -1}{2}\right)}{\Gamma \left(\frac{\nu }{2}\right)}-\frac{\alpha \nu }{\nu -2}+\frac{\alpha^{2}}{2}{\left(\frac{\nu }{2}\right)}^{3/2}\frac{\Gamma \left(\frac{\nu -3}{2}\right)}{\Gamma \left(\frac{\nu }{2}\right)}\sqrt{\frac{2}{\pi }}\left(\frac{3}{1+s^{2}}\delta +2\delta^{3}\right),\\ M_{2}\left(\alpha ,s,\nu \right)= & \frac{\nu }{\nu -2}-\alpha {\left(\frac{\nu }{2}\right)}^{3/2}\frac{\Gamma (\frac{\nu -3}{2})}{\Gamma (\frac{\nu }{2})}\sqrt{\frac{2}{\pi }}\left(\frac{3}{1+s^{2}}\delta +2\delta^{3}\right)+\frac{3\alpha^{2}\nu^{2}}{2(\nu -2)(\nu -4)},\\ M_{3}\left(\alpha ,s,\nu \right)= & {\left(\frac{\nu }{2}\right)}^{3/2}\frac{\Gamma \left(\frac{\nu -3}{2}\right)}{\Gamma \left(\frac{\nu }{2}\right)}\sqrt{\frac{2}{\pi }}\left(\frac{3}{1+s^{2}}\delta +2\delta^{3}\right)-\frac{3\alpha \nu^{2}}{\left(\nu -2)(\nu -4\right)}\\ \; & +\frac{\alpha^{2}}{2}{\left(\frac{\nu }{2}\right)}^{5/2}\frac{\Gamma \left(\frac{\nu -5}{2}\right)}{\Gamma \left(\frac{\nu }{2}\right)}\sqrt{\frac{2}{\pi }}\left(\frac{15}{{\left(1+s^{2}\right)}^{2}}\delta +\frac{20}{1+s^{2}}\delta^{3}+8\delta^{5}\right),\\ M_{4}\left(\alpha ,s,\nu \right)= & \frac{3\nu^{2}}{\left(\nu -2)(\nu -4\right)}-\alpha {\left(\frac{\nu }{2}\right)}^{5/2}\frac{\Gamma \left(\frac{\nu -5}{2}\right)}{\Gamma \left(\frac{\nu }{2}\right)}\sqrt{\frac{2}{\pi }}\left(\frac{15}{{\left(1+s^{2}\right)}^{2}}\delta +\frac{20}{1+s^{2}}\delta^{3}+8\delta^{5}\right)\\ \; & +\frac{15\alpha^{2}\nu^{3}}{2(\nu -2)(\nu -4)(\nu -6)}. \end{array}$$

### 2.4. Stochastic Representation the GAST Distribution

Altun (2018) [8] provided a stochastic representation of X∼GAST(α,s,ν) as follows.

**Theorem** **1.** *If the random variables W∼AST(α,ν) and Z∼t(ν+1) are independent, then we have*

(26) $$W\left|\left\{\sqrt{\frac{1+\nu }{W^{2}+\nu }}sW>Z\right.\right\}∼GAST\left(\alpha ,s,\nu \right)$$

According to (26) given by [8] , we can generate random samples from the GAST distribution by the following procedure:

**Step 1.**  Generate W∼AST(α,ν) and Z∼t(ν+1).
**Step 2.**  If $\sqrt{\left(1+\nu \right)/\left(W^{2}+\nu \right)}sW>Z$, then keep *W*. Otherwise, go to Step 1.

## 3. Parameter Estimation

In parameter estimation, the maximum likelihood estimation has been widely utilized because of its transitivity. Let $x=\{x_{1},x_{2},\ldots ,x_{n}\}$ be a random sample from the GAST(x;α,s,ν) distribution. The log-likelihood function is given by

(27) $$\begin{array}{cc} \ell (\alpha ,s,\nu |x)=\; & \sum_{i=1}^{n}\log\left[\frac{{\left(1-\alpha x_{i}\right)}^{2}+1}{c(\alpha ,s,\nu )}\right]+\sum_{i=1}^{n}\log\left[t(x_{i};\nu )\right]+\\ \; & \sum_{i=1}^{n}\log\left[T\left(\sqrt{\frac{1+\nu }{x_{i}^{2}+\nu }}sx_{i};\nu +1\right)\right]. \end{array}$$

By taking the partial derivatives with respect to α,s and ν, we have

(28) $$\begin{array}{cc} \frac{\partial \ell }{\partial \alpha } & =\sum_{i=1}^{n}\frac{\left(-2x_{i}\right)\left(1-\alpha x_{i}\right)c\left(\alpha ,s,\nu \right)-c_{\alpha }\left(\alpha ,s,\nu \right)\left[{\left(1-\alpha x_{i}\right)}^{2}+1\right]}{\left[{\left(1-\alpha x_{i}\right)}^{2}+1\right]c\left(\alpha ,s,\nu \right)},\\ \frac{\partial \ell }{\partial s} & =n\frac{-c_{s}\left(\alpha ,s,\nu \right)}{c(\alpha ,s,\nu )}+\sum_{i=1}^{n}x_{i}\sqrt{\frac{\nu +1}{x_{i}^{2}+\nu }}\omega_{i}^{*},\\ \frac{\partial \ell }{\partial \nu } & =n\frac{-c_{\nu }\left(\alpha ,s,\nu \right)}{c(\alpha ,s,\nu )}+\sum_{i=1}^{n}\tau_{i}^{*}+\sum_{i=1}^{n}\frac{sx_{i}\left(x_{i}^{2}-1\right)}{2\sqrt{\frac{\nu +1}{x_{i}^{2}+\nu }}{\left(x_{i}^{2}+\nu \right)}^{2}}\omega_{i}^{*}, \end{array}$$

where

$$\omega_{i}^{*}=\frac{t\left(\sqrt{\frac{1+\nu }{x_{i}^{2}+\nu }}sx_{i};\nu +1\right)}{T\left(\sqrt{\frac{1+\nu }{x_{i}^{2}+\nu }}sx_{i};\nu +1\right)},\;\tau_{i}^{*}=\frac{t_{\nu }\left(x_{i};\nu \right)}{t(x_{i};\nu )}.$$

Remark that $c_{\alpha }\left(\alpha ,s,\nu \right)$, $c_{s}\left(\alpha ,s,\nu \right)$, $c_{\nu }\left(\alpha ,s,\nu \right)$, and $t_{\nu }\left(x_{i};\nu \right)$ are the partial derivatives of c(α,s,ν) and $t(x_{i};\nu )$. The solution $\left(\hat{\alpha },\hat{s},\hat{\nu }\right)$ satisfying $\frac{\partial \ell }{\partial \alpha }=0$, $\frac{\partial \ell }{\partial s}=0$, $\frac{\partial \ell }{\partial \nu }=0$ at the same time is the MLE of (α,s,ν). To solve the system of nonlinear equations in (28), a numerical method is required. In the following subsections, we introduce the algorithm for solving MLE: L-BFGS-B (Byrd et al., 1995 [24]) in Section 3.1. In order to improve estimation accuracy by enhancing sample representativeness, we incorporate a non-parametric quantile estimation method (Harrell and Davis 1982 [25]) introduced in Section 3.2. In Section 3.3, we evaluate the effectiveness of the algorithm and quantile estimation method by simulation. In our study, we use R software version 4.4.1 to conduct simulation.

### 3.1. L-BFGS-B

L-BFGS-B (Byrd et al., 1995 [24]) is a limited-memory algorithm for solving large nonlinear optimization problems subject to simple bounds on the variables. The essence of the algorithm is a quasi-Newton method. At each iteration, a limited-memory BFGS approximation to the Hessian matrix is updated. This limited-memory matrix is used to define a quadratic model of the objective function, in our study indicating (27). Given a set of samples $x={\left\{x_{i}\right\}}_{i=1}^{n}$, the optimization problem can be formulated as follows:

$$\max_{\alpha ,s,\nu }\ell \left(\alpha ,s,\nu |x\right).$$

We summarize the procedures of L-BFGS-B as following Algorithm 1.
**Algorithm 1** L-BFGS-B for MLE1:**Input:** Initial guesses for parameters $\alpha_{0},s_{0},\nu_{0}$, tolerance ϵ, maximum number of iterations *N*, bounds $\left(\alpha_{\min},s_{\min},\nu_{\min}\right)$ and $\left(\alpha_{\max},s_{\max},\nu_{\max}\right)$2:**Output:** Estimated parameters $\hat{\alpha },\hat{s},\hat{\nu }$3:Initialize k←04:Initialize parameters $\theta^{\left(0\right)}\leftarrow \left(\alpha_{0},s_{0},\nu_{0}\right)$5:**repeat**6:   Compute the gradient $\nabla \ell (\theta^{\left(k\right)})$7:   Compute the search direction $p^{\left(k\right)}$ using a two-stage approach [24]8:   Project the search direction $p^{\left(k\right)}$ to satisfy the bounds9:   Line search: find step size $\lambda^{\left(k\right)}$ that maximizes $\ell (\theta^{\left(k\right)}+\lambda^{\left(k\right)}p^{\left(k\right)})$10:   Update parameters: $\theta^{\left(k+1\right)}\leftarrow \theta^{\left(k\right)}+\lambda^{\left(k\right)}p^{\left(k\right)}$11:   k←k+112:**until** $∥\nabla \ell (\theta^{\left(k\right)})∥<\epsilon$ or k≥N13:$\left(\hat{\alpha },\hat{s},\hat{\nu }\right)\leftarrow \theta^{\left(k\right)}$

We chose the L-BFGS-B algorithm because the degree of freedom ν must be greater than 2 for the GAST distribution. If the unconstrained optimization method is used, missing values are likely to appear in the optimization process.

### 3.2. QMC-MLE

In this subsection, we introduce a method for improving the accuracy of MLE. It is well-known that the accuracy of MLE depends on the sample size to a certain extent. If the sample misses the turning points of the population density, it is less representative, which may lead to lower estimation accuracy. This situation is prone to occur in small sized samples and especially bimodal cases. Fang and Wang (1994) [12] pointed out that the set of equal quantiles $\left\{p_{i}=\left(2i-1\right)/2n,i=1,\ldots ,n\right\}$ has the best representativeness in the sense of *F*-discrepancy. In Section 1, we introduce a QMC method to generate the RPs of a distribution with known parameters. However, for a distribution with unknown parameters, how can we obtain the $p^{th}$ quantile of the distribution *F*? Harrell and Davis (1982) [25] proposed a distribution-free method: the Harrell–Davis (HD) quantile estimator. We use this estimator to calculate the set of equal quantiles of *F*, and then substitute these *n* quantiles into the likelihood function ℓ(θ∣x) for calculation. Li and Fang (2024) [20] called the MLE method with HD quantile estimator as QMC-MLE, presented below.

Let $x=\{x_{1},\ldots ,x_{n}\}$ be a random sample of size *n* from the GAST distribution. Denote $X_{\left(i\right)}$ as the $i^{th}$ largest value in x and $F^{-1}\left(p\right)$ as the $p^{th}$ population quantile.

**Step 1:**  Generate a set of points uniformly scattered on (0,1) through
  $$p_{i}=\frac{2i-1}{2n},\;i=1,\ldots ,n.$$
**Step 2:**  Use the Harrell–Davis quantile estimator to process sample:
  $$Q\left(p_{i}\right)=\sum_{i=1}^{n}W_{n,i}X_{\left(i\right)},$$
  where
  $$\begin{array}{cc} W_{n,i} & =\frac{1}{\beta \{\left(n+1\right)p_{i},\left(n+1\right)\left(1-p_{i}\right)\}}\int_{(i-1)/n}^{i/n}y^{\left(n+1\right)p_{i}-1}{\left(1-y\right)}^{\left(n+1\right)\left(1-p_{i}\right)-1}\;dy\\ \; & =I_{i/n}\left\{p_{i}\left(n+1\right),\left(1-p_{i}\right)\left(n+1\right)\right\}-I_{(i-1)/n}\left\{p_{i}\left(n+1\right),\left(1-p_{i}\right)\left(n+1\right)\right\}, \end{array}$$
  and $I_{x}\left\{a,b\right\}$ denotes the incomplete beta function.
**Step 3:**  Let $z_{i}=Q\left(p_{i}\right)$, for i=1,…,n. Therefore, the $x=(x_{1},\ldots ,x_{n})$ in the log-likelihood function is replaced by $z=(z_{1},\ldots ,z_{n})$ such that the objective function based on the revised sample is
  (29) $$\ell \left(\theta |z\right)=\sum_{i=1}^{n}\ln\left(f(z_{i};\alpha ,s,\nu )\right).$$
**Step 4:**  Use the L-BFGS-B algorithm to find the MLE of θ by maximizing (29).

### 3.3. Simulation

Before the simulation, we introduce four measures of the estimation accuracy: L2.pdf, L2.cdf, absolute bias index (ABI) and Kullback–Leibler (KL) divergence. Denote the true underlying distribution as *F* in cdf or *f* in pdf, and the estimated distribution as $\hat{F}$ or $\hat{f}$. The four measures are defined as follows:

- **L2.pdf** between two densities is defined as
  $$L_{2}\left(f,\hat{f}\right)={\left[\int_{-\infty }^{\infty }{\left(f\left(x\right)-\hat{f}\left(x\right)\right)}^{2}dx\right]}^{1/2};$$
- **L2.cdf** between two cdf’s is defined as
  $$L_{2}\left(F,\hat{F}\right)={\left[\int_{-\infty }^{\infty }{\left(F\left(x\right)-\hat{F}\left(x\right)\right)}^{2}dx\right]}^{1/2};$$
- **Absolute bias index** (ABI) is used to evaluate the overall estimation bias in parameters in which $\hat{\mu }$ and $\hat{\sigma }$ denote the estimated expectation and standard deviation of the GAST distribution, defined as
  $$ABI=\frac{1}{2}\left(\left|\frac{\mu -\hat{\mu }}{\mu }\right|+\left|\frac{\sigma -\hat{\sigma }}{\sigma }\right|\right);$$
- **Kullback–Leibler (KL)** divergence or the so-called relative entropy is used to measure the difference from one probability distribution to another, defined as follows:
  $$D_{KL}\left(F||\hat{F}\right)=\int_{-\infty }^{\infty }f\left(x\right)\log\left(\frac{f(x)}{\hat{f}\left(x\right)}\right)dx.$$

In the simulation, we generate samples by the inverse transformation method and mainly focus on the small sample case. To study both unimodal and bimodal cases, we choose five parameter settings, No.VII, VIII, IX, X and XI, of the GAST distribution from Figure 1 as the underlying distributions, among which the No.VII, VIII, and XI distributions are bimodal. The sample size *n* is set to be 25,50,100 and 300. After N=100 times of repetition, the average of $\left(\hat{\alpha },\hat{s},\hat{\nu }\right)$ is set to be the parameters of the estimated GAST distribution. The precision of the estimates is evaluated by L2.pdf, L2.cdf, ABI and KL, summarized in Table 2, in which “plain” indicates the MLE resulting from the original sample $x=(x_{1},\ldots ,x_{n})$, and “qmc” uses the revised sample $z=(z_{1},\ldots ,z_{n})$.

The best performance in the sense of each measure for each pair of distribution type and sample size is highlighted in bold in Table 2. The QMC-MLE method performs better than the plain MLE in most cases, especially for the No.VIII, IX and X distributions. However, for the No.VII and XI distributions, the QMC-MLE has no obvious advantage. The No.IX and X distributions are unimodal, but the No.VIII is bimodal. From the pdf plot of No.VIII distribution, we can see that although it is bimodal, its first peak is not as obvious as the peaks of No.VII and XI distributions. In the pdf plots of No.VII and XI distributions, as *x* increases, the density function experiences a steep decline after the first peak, while for the No.VIII distribution, the decline lasts only for a short distance before it begins to rise again. Therefore, we have reasons to believe that the QMC-MLE method is more suitable for unimodal functions or bimodal functions of which one peak is not obvious.

In addition, for No.XI GAST distribution, in the sense of KL divergence, the plain MLE is better than the QMC-MLE for all sample sizes. As for the No.XI case under other measures, although the QMC-MLE performs better when n=25 and 50, it becomes less effective for n=100 and 300, which may be caused by the consistency of MLE. According to the discussion above, when we conduct case studies in Section 5, the QMC-MLE will be only used for unimodal samples in parameter estimation, while for bimodal samples, we will use the plain MLE. Nevertheless, this simulation study reveals that the MLE method (both plain and QMC) is appropriate for estimating the GAST parameters due to the small values of four bias measurements.

## 4. RPs of the GAST Distribution

Recall that in Section 1, we introduced three types of representative points: MC-RPs, QMC-RPs, and MSE-RPs. In this section, we will find these three types of RPs of the GAST distribution for different sample sizes *n*, and use them to estimate moments and densities in Section 4.1 and Section 4.2, respectively.

### 4.1. Moment Estimation

For a given *n*, MC-RPs will be generated by the inverse transformation method. QMC-RPs can be easily obtained by (6) while MSE-RPs are calculated through a parametric k-means algorithm proposed by Stampfer and Stadlober (2002) [21]. We summarize the computation procedure of the k-means algorithm for approximating MSE-RPs of the GAST distribution as follows.

**Step 1:**  For a given pdf f(x;α,s,ν), the number of RPs: *n*, and t=0, input a set of initial points $b_{1}^{\left(t\right)}<b_{2}^{\left(t\right)}<\cdots <b_{n}^{\left(t\right)}$. Here we take *n* QMC-RPs as the initial values. Determine a partition of R as
  $$I_{i}^{\left(t\right)}=\left(a_{i-1}^{\left(t\right)},a_{i}^{\left(t\right)}\right],\;i=1,\ldots ,n-1,\;I_{n}^{\left(t\right)}=\left(a_{n-1}^{\left(t\right)},a_{n}^{\left(t\right)}\right),$$
  where
  $$a_{0}^{\left(t\right)}=-\infty ,\;a_{i}^{\left(t\right)}=\left(b_{i-1}^{\left(t\right)}+b_{i}^{\left(t\right)}\right)/2,\;i=1,\ldots ,n-1,\;a_{n}^{\left(t\right)}=\infty .$$
**Step 2:**  Calculate probabilities
  $$p_{j}^{\left(t\right)}=\int_{I_{j}^{\left(t\right)}}f\left(x;\alpha ,s,\nu \right)dx,\;j=1,\ldots ,n;$$
  and the condition means
  $$b_{j}^{\left(t+1\right)}=\frac{\int_{I_{j}^{\left(t\right)}}xf\left(x;\alpha ,s,\nu \right)dx}{\int_{I_{j}^{\left(t\right)}}f\left(x;\alpha ,s,\nu \right)dx}=\frac{\int_{I_{j}^{\left(t\right)}}xf\left(x;\alpha ,s,\nu \right)dx}{p_{j}^{\left(t\right)}}.$$
**Step 3:**  If two sets, $\left\{b_{j}^{\left(t\right)}\right\}$ and $\left\{b_{j}^{\left(t+1\right)}\right\}$ are identical, the process stops and the outputs $\left\{b_{j}^{\left(t\right)}\right\}$ as the MSE-RPs of the distribution with probabilities $\left\{p_{j}^{\left(t\right)}\right\}$. Otherwise, let t:=t+1 and go back to **Step 1**.

Let *Y* be a discrete distribution with probability mass function $P\left(Y=b_{j}\right)=p_{j},$j=1,…,n, which is an approximate distribution to the GAST distribution. Then, the estimates of mean, variance, skewness and kurtosis can be calculated by

(30) $$\begin{array}{ccc} \; & E\left[Y\right]=\sum_{j=1}^{n}b_{j}p_{j}=\mu_{Y}, & Var\left[Y\right]=\sum_{j=1}^{n}{\left(b_{j}-\mu_{Y}\right)}^{2}p_{j}=\sigma_{Y}^{2},\\ \; & Sk\left[Y\right]=\frac{1}{\sigma_{Y}^{3}}\sum_{j=1}^{n}{\left(b_{j}-\mu_{b}\right)}^{3}p_{j}, & Ku\left[Y\right]=\frac{1}{\sigma_{Y}^{4}}\sum_{j=1}^{n}{\left(b_{j}-\mu_{Y}\right)}^{4}p_{j}-3. \end{array}$$

We use the No.IX, X and XI as the underlying distributions and consider n=10,20,30. It is clear that MC-RPs are random samples of size *n*. For fair comparisons, we generate *N* samples of size *n* and then take the average of the estimated statistics as the results of the MC(N) method. In our study, we choose N=10,100. The true parameters and four statistics of the three underlying distribution are listed in Table 3. The bias of the estimated results is summarized in Table 4, Table 5 and Table 6.

The results indicate that the estimates based on MSE-RPs perform the best for all underlying distributions and sample sizes. The performance of MC-RPs is unstable. Sometimes the average estimates of moments based on MC-RPs are more accurate than those based on QMC-RPs, but in general, appear less effective. In addition, we can observe that with the increase in the number *n*, the overall effect of estimation is better. The estimates of higher-order moments (skewness and kurtosis) are worse than those of lower-order moments (mean and variance).

### 4.2. Kernel Density Estimation

Another application of representative points is density estimation. In the field of signal transmission, the input signal is often converted into discrete data in the transmitter and then reconstructed in the receiver. For a distribution with unknown parameters, how do we use a set of data to construct its overall density function? Here, we introduce a kernel estimation method proposed by Rosenblatt (1956) [22] and Parzen (1962) [26]. Given a fixed number of points $\left\{x_{1},\ldots ,x_{n}\right\}$ from the original signal, the density estimation of f(x) is given by

$${\hat{f}}_{h}\left(x\right)=\frac{1}{n}\sum_{i=1}^{n}k_{h}\left(x-x_{i}\right)=\frac{1}{nh}\sum_{i=1}^{n}k\left(\frac{x-x_{i}}{h}\right),$$

where k(·) is the kernel function is the bandwidth and $k_{h}\left(y\right)=\frac{1}{h}k\left(\frac{y}{h}\right)$. The most popular kernel is the standard normal density function

$$k\left(x\right)=\phi \left(x\right)=\frac{1}{\sqrt{2\pi }}e^{-\frac{1}{2}x^{2}}.$$

In our study, we employ the representative points $\left\{b_{1},\ldots ,b_{n}\right\}$ from the GAST distribution as the samples with their corresponding probabilities $p_{i},i=1,\ldots ,n$. The density estimation of f(x) can be extended to

$${\hat{f}}_{h}\left(x\right)=\sum_{i=1}^{n}k_{h}\left(x-x_{i}\right)p_{i}=\frac{1}{h}\sum_{i=1}^{n}k\left(\frac{x-x_{i}}{h}\right)p_{i}.$$

The choice of the bandwidth *h* is very important. Here, we set a search range {0.05,0.06,…,1} for *h*. In the following comparisons, we utilize three types of RPs having sample sizes n=10,20,30 for the kernel density estimation of No.IX, X and XI distributions, and evaluate the performances by the minimum L2.pdf between ${\hat{f}}_{h}\left(x;\alpha ,s,\nu \right)$ and f(x;α,s,ν).

**Figure 2:**
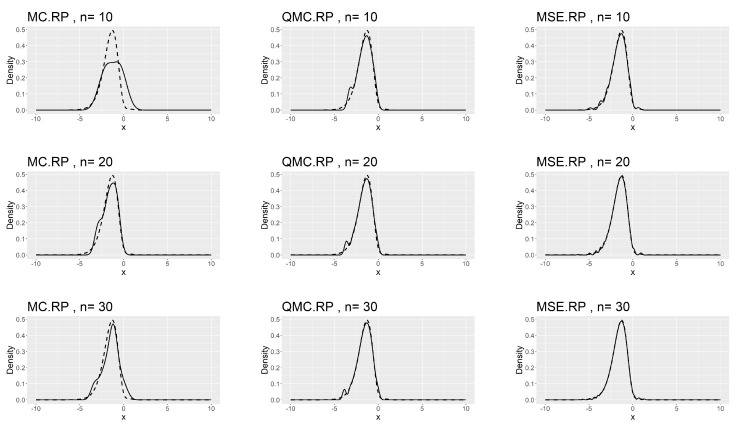
Comparing plots of the fitted densities (in solid lines) by kernel density estimation and the true densities (in dashed lines) for the No.IX distribution.

**Figure 3:**
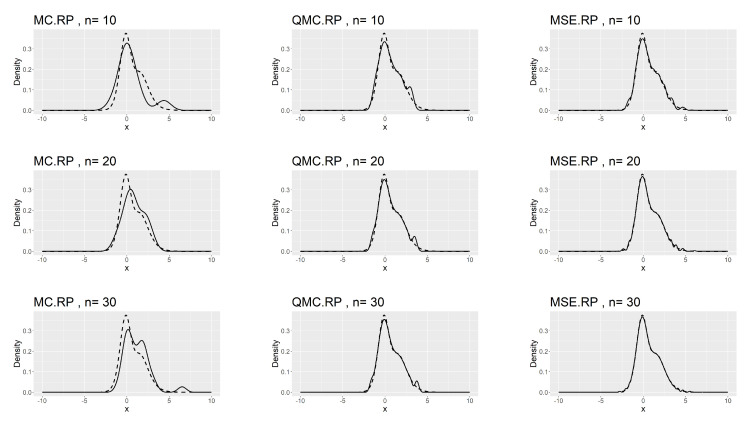
Comparing plots of the fitted densities (in solid lines) by kernel density estimation and the true densities (in dashed lines) for the No.X distribution.

**Figure 4:**
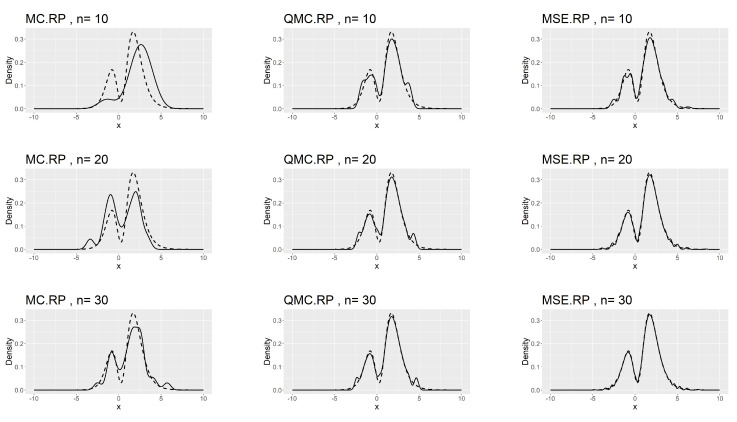
Comparing plots of the fitted densities (in solid lines) by kernel density estimation and the true densities (in dashed lines) for the No.XI distribution.

Table 7, Table 8 and Table 9 show that the kernel density estimation based on MSE-RPs always has the minimum L2.pdf, which decreases as *n* increases. For the underlying distribution No.IX, we notice that the minimum L2.pdf based on the MSE-RPs with size 10 is only 0.0306, which is even smaller than that based on the QMC-RPs with size 30 (0.0341). Figure 2, Figure 3 and Figure 4 show the comparing fitting plots of different sets of representative points. It is obvious that the fitting effect increases with *n*, and the MSE-RPs-based kernel estimation has the best fitting effect, followed by the QMC-RPs-based estimation. It is worth mentioning that for the MC-RPs-based density estimation, due to the randomness of the Monte Carlo method, the density curve fitted out each time differs greatly, and in many cases, it is not sufficient to reconstruct the original density function.

## 5. Case Studies

In this section, we will utilize three types of RPs to study real data samples. Before calculating the RPs, we incorporate two additional parameters in the GAST distribution, the location parameter μ and the scale parameter σ, to fit the samples. The pdf is given by

$$f\left(x;\alpha ,s,\nu ,\mu ,\sigma \right)=\frac{{\left(1-\alpha \left(\frac{x-\mu }{\sigma }\right)\right)}^{2}+1}{\sigma c(\alpha ,s,\nu )}t\left(\frac{x-\mu }{\sigma };\nu \right)T\left(\sqrt{\frac{1+\nu }{{\left(\frac{x-\mu }{\sigma }\right)}^{2}+\nu }}s\left(\frac{x-\mu }{\sigma }\right);\nu +1\right),$$

where c(α,s,ν) is the same as that in Formula (11). For the sample data, we choose both unimodal and bimodal types, which are the $O_{3}$ data and the Faithful Geyser data.

### 5.1. $O_{3}$ Data

These data are from the website (https://archive.ics.uci.edu/dataset/360/air+quality (accessed on 15 September 2024)), which contains hourly averaged responses from an Air Quality Chemical Multisensor Device in an Italian city. We selected the “PT08.S5($O_{3}$)” (denoted as “$O_{3}$” in this article) data as the study object. After setting the interception time from September 1 to November 30 in 2004, and removing the missing values, we derive 90 observations. We summarize the parameter estimation results of GAST(α,s,ν,μ,σ) obtained by the QMC-MLE in Table 10, providing the estimated GAST model as follows

(31) GAST(−0.1518,−0.2030,16.9607,1219.228,385.0162).

**Figure 5:**
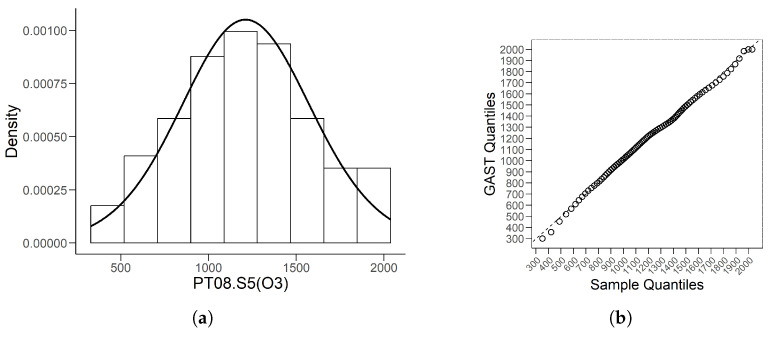
(**a**) is the histogram of $O_{3}$ data with fitted GAST density line. (**b**) is the associated QQ plot by HD quantile estimator.

We present the histogram with the fitted density for $O_{3}$ data in Figure 5a. After calculating the $\left\{p_{i}=\left(2i-1\right)/2n,i=1,\ldots ,n\right\}$ quantiles of these data by the HD quantile estimator introduced in Section 3.2, we obtain the associated QQ plot given in Figure 5b. Figure 5 shows the good fitting effect of the GAST model on this unimodal data.

The mean, variance, skewness, kurtosis of the distribution (31) are as follows

(1209.544,168359.9,0.0362,0.601).

We generate MC-RPs, QMC-RPs and MSE-RPs of size 30, from the GAST model (31) using the methods discussed in Section 4.1. Table 11 summarizes the bias of the estimation of the four statistics based on the MC, QMC, and MSE methods.

Although the bias of the estimated variance in Table 11 is large in value, it is relatively small compared to the true variance of the model, which is 168,359.9. As shown in Table 11, the MSE-RPs estimate the moments of the model more accurately than the other two types of RPs.

**Figure 6:**
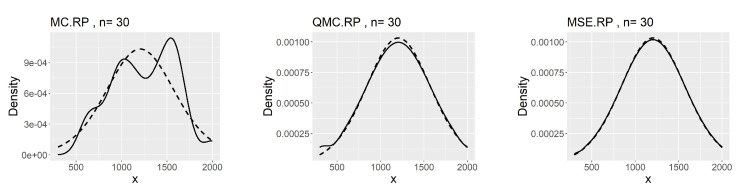
Comparing plots of the fitted densities (in solid lines) by kernel density estimation and the density of the distribution (31).

The comparisons of the kernel density estimates based on MC, QMC, and MSE RPs are presented in Figure 6.

The corresponding minimum L2.pdf’s between the kernel estimates and the density of the model (31) are 0.00237 for MC, 0.00026 for QMC and 0.00025 for MSE. As shown in Figure 6, although the estimated kernel density based on the QMC method is well-fitted, it is not as good as that based on the MSE method at the beginning and at the peak.

### 5.2. Faithful Geyser Data

The Faithful Geyser Data, a commonly used dataset in R software, is a record of the waiting time between eruptions and the duration time of these eruptions for Old Faithful Geyser in Yellow National Park, Wyoming, USA. In this study, we use the waiting-time samples which include 299 observations.

Since these data are bimodal, we use the plain-MLE to estimate parameters. The results are given in the Table 12, providing the GAST model as

(32) GAST(−2.4016,−0.2322,100,70.6301,8.7872).

**Figure 7:**
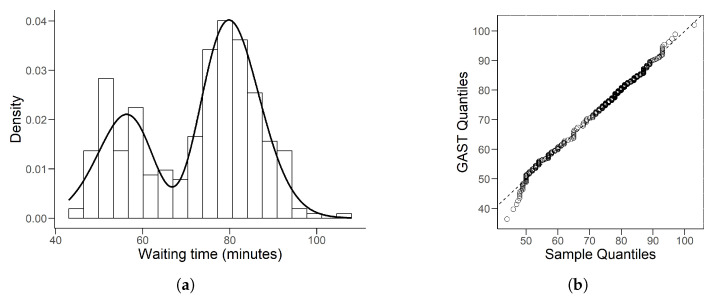
(**a**) is the histogram of Faithful Geyser data with fitted GAST density line. (**b**) is the associated QQ plot by traditional quantile estimator.

The histogram with the fitted density for Faithful Geyser data is given in Figure 7a. Denoting $X_{\left(1\right)}<\ldots <X_{\left(n\right)}$ as the order statistics of this bimodal data, we calculate its quantiles by the traditional estimator:

$$Q_{p}=\left(1-g\right)X_{\left(j\right)}+gX_{\left(j+1\right)},$$

where (n+1)p=j+g and *j* is the integral part of (n+1)p. The associated QQ plot is given in Figure 7b.

Table 12 shows that the estimated ν is 100, which is the upper bound we set. From this point of view, we can assume that ν→∞ in this fitting model. As described in Section 2.1, this model is actually a subdistribution of the GAST: ASN, indicating that the GAST model is flexible since it can adapt to different types of data. From the QQ plot in Figure 7, we notice that when data are less than 50, the scatter point deviates far from the line, which can also be observed from Figure 7a. The fitting curve rises slowly at the beginning, so the sample quantiles will be larger than the GAST quantiles. When the data are greater than 50, where more samples are located, this distribution fits the data well. Hence, the estimated GAST model (32) is still acceptable.

The mean, variance, skewness, and kurtosis of the distribution (32) are

(72.3205,192.9088,−0.4496,−0.8031).

We generate MC-RPs, QMC-RPs and MSE-RPs of size 50 from the model (32). The estimation biases of the four statistics are summarized in Table 13.

**Figure 8:**
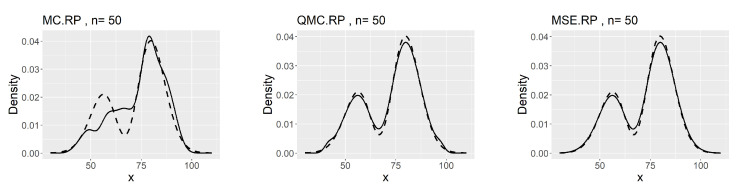
Comparing plots of the fitted densities (in solid lines) by kernel density estimation and the density of the distribution (32).

We observe that the MSE-RPs have the same mean as the population expectation, which is described in (9). Compared to MC-RPs and QMC-RPs, the MSE method estimates the moments of the model more accurately. The comparison of the kernel density plots is presented in Figure 8.

The corresponding minimum L2.pdf’s between the kernel estimates and the density of the model (32) are 0.0041 for MC, 0.0029 for QMC and 0.0027 for MSE. The MSE-RPs still perform the best.

## 6. Conclusions

This paper mainly studies different types of representative points of the GAST distribution and the applications of these RPs. The comparative analyses across various sample sizes and both unimodal and bimodal GAST distributions reveal that the RPs obtained by the MSE method consistently outperform the others in the applications of estimating moments and densities. However, the performance on estimating higher-order moments, such as skewness and kurtosis, shows the limitations of RPs on capturing higher-order statistical properties. Therefore, the number of RPs *n* must adopt a larger value to reduce the bias of higher-order moment estimation. This paper also incorporates QMC-MLE for parameter estimation of the GAST distribution. For unimodal or bimodal data with an unclear peak, the QMC-MLE method improves parameter estimation accuracy. However, in bimodal cases, plain MLE is more effective. Combined with such property, we can model different types of data accordingly.

## Figures and Tables

**Table 1:** The parameter settings according to a uniform design table $U_{16}\left(16^{3}\right)$.

No.	α	*s*	ν	No.	α	*s*	ν
**I**	−2.4	1.6	9	**IX**	3.2	−2	16
**II**	−0.8	0.4	3	**X**	1.2	1.2	15
**III**	−1.6	−1.6	6	**XI**	3.6	0.8	11
**IV**	1.6	−2.4	4	**XII**	0.4	0	18
**V**	−1.2	2.4	17	**XIII**	0.8	−1.2	10
**VI**	2.4	−0.4	8	**XIV**	0	3.2	7
**VII**	−2	−0.8	14	**XV**	−0.4	−2.8	12
**VIII**	2.8	2	5	**XVI**	2	2.8	13

**Table 2:** The comparisons between the plain MLE and QMC-MLE in four measures (n=25,50,100,300; N=100).

No.	Method	*n* = 25				*n* = 50				*n* = 100				*n* = 300			
		L2.pdf	L2.cdf	ABI	KL	L2.pdf	L2.cdf	ABI	KL	L2.pdf	L2.cdf	ABI	KL	L2.pdf	L2.cdf	ABI	KL
VII	plain	**0.0326**	0.0469	0.0730	**0.0039**	**0.0133**	**0.0153**	**0.0207**	**0.0012**	**0.0064**	**0.0131**	**0.0260**	**0.0001**	**0.0041**	**0.0056**	0.0098	**0.0001**
VII	qmc	0.0446	**0.0440**	**0.0552**	0.0079	0.0201	0.0218	0.0307	0.0019	0.0163	0.0224	0.0326	0.0008	0.0076	0.0071	**0.0078**	0.0002
VIII	plain	0.0896	0.1161	0.1822	0.0897	0.0575	0.0721	0.1026	0.0256	0.0178	0.0358	0.0809	0.0024	0.0077	0.0186	0.0436	0.0005
VIII	qmc	**0.0670**	**0.0923**	**0.1590**	**0.0830**	**0.0359**	**0.0496**	**0.0749**	**0.0181**	**0.0127**	**0.0286**	**0.0650**	**0.0019**	**0.0058**	**0.0160**	**0.0346**	**0.0004**
IX	plain	**0.0317**	0.0365	0.0321	**0.0073**	0.0185	0.0214	0.0230	0.0034	0.0110	0.0129	0.0172	0.0021	0.0047	0.0049	0.0092	**0.0003**
IX	qmc	0.0302	**0.0363**	**0.0306**	0.0074	**0.0108**	**0.0150**	**0.0160**	**0.0026**	**0.0056**	**0.0082**	**0.0101**	**0.0015**	**0.0029**	**0.0035**	**0.0057**	0.0004
X	plain	**0.0691**	0.0762	0.1443	**0.0097**	0.0453	0.0525	0.0967	0.0056	0.0172	0.0261	0.0525	0.0010	0.0066	0.0125	0.0272	0.0003
X	qmc	0.0570	**0.0663**	**0.1293**	0.0104	**0.0381**	**0.0476**	**0.0886**	**0.0041**	**0.0112**	**0.0207**	**0.0372**	**0.0003**	**0.0058**	**0.0113**	**0.0203**	**0.0002**
XI	plain	0.0689	0.0883	0.1073	**0.0194**	0.0398	0.0487	0.0576	**0.0031**	**0.0134**	**0.0151**	0.0265	**0.0067**	0.0058	**0.0063**	0.0087	**0.0005**
XI	qmc	**0.0563**	**0.0552**	**0.1043**	0.0263	**0.0390**	**0.0342**	**0.0525**	0.0104	0.0279	0.0265	**0.0196**	0.0104	**0.0041**	0.0127	**0.0069**	0.0010

**Table 3:** True parameters and statistics of the underlying distributions.

No.	α	*s*	ν	E(X)	Var(X)	Sk(X)	Ku(X)
IX	3.2	−2	16	−1.5997	0.7971	−0.7117	1.4857
X	1.2	1.2	15	0.6272	1.7074	0.6258	0.3101
XI	3.6	0.8	11	1.2301	3.0124	−0.2293	0.0633

**Table 4:** Estimation bias of four statistics for the No.IX distribution GAST(3.2,−2,16).

Statistics	Category	10	20	30
Mean	MC(10)	−0.1180	−0.1562	−0.0149
	MC(100)	−0.0291	0.0214	0.0132
	QMC	0.0090	0.0031	0.0014
	MSE	**0.0000**	**0.0000**	**0.0000**
Variance	MC(10)	0.1194	0.3288	0.0769
	MC(100)	−0.0024	−0.0212	−0.0044
	QMC	−0.1455	−0.0889	−0.0668
	MSE	**−0.0231**	**−0.0063**	**−0.0029**
Skewness	MC(10)	0.3486	0.1735	0.1472
	MC(100)	0.3667	0.2676	0.2479
	QMC	0.2740	0.1677	0.1227
	MSE	**0.0259**	**0.0045**	**0.0027**
Kurtosis	MC(10)	−2.4314	−1.3967	−0.7850
	MC(100)	−2.3704	−1.6857	−1.3343
	QMC	−2.0406	−1.6037	−1.3769
	MSE	**−0.5136**	**−0.1855**	**−0.0942**

The best performance within each statistic per sample size is highlighted in bold.

**Table 5:** Estimation bias of four statistics for the No.X distribution GAST(1.2,1.2,15).

Statistics	Category	10	20	30
Mean	MC(10)	−0.0472	0.0170	0.1001
	MC(100)	0.0736	0.0219	−0.0217
	QMC	−0.0102	−0.0051	−0.0035
	MSE	**0.0000**	**0.0000**	**0.0000**
Variance	MC(10)	−0.0684	−0.1739	−0.1644
	MC(100)	0.0757	0.1530	0.0124
	QMC	−0.2179	−0.1213	−0.0861
	MSE	**−0.0394**	**−0.0109**	**−0.0051**
Skewness	MC(10)	−0.6435	−0.1809	−0.0803
	MC(100)	−0.2718	−0.1878	−0.1476
	QMC	−0.1634	−0.1104	−0.0873
	MSE	**−0.0106**	**−0.0034**	**−0.0017**
Kurtosis	MC(10)	−1.4404	−0.5846	−0.5425
	MC(100)	−1.3711	−0.8694	−0.6661
	QMC	−1.0604	−0.7846	−0.6516
	MSE	**−0.2881**	**−0.1005**	**−0.0514**

The best performance within each statistic per sample size is highlighted in bold.

**Table 6:** Estimation bias of four statistics for the No.XI distribution GAST(3.6,0.8,11).

Statistics	Category	10	20	30
Mean	MC(10)	0.0529	−0.1639	0.0381
	MC(100)	−0.0581	−0.0778	0.0276
	QMC	−0.0189	−0.0082	−0.0032
	MSE	**0.0000**	**0.0000**	**0.0000**
Variance	MC(10)	0.4662	0.1453	−0.3346
	MC(100)	0.2882	−0.0598	−0.0723
	QMC	−0.3569	−0.2171	−0.1635
	MSE	**−0.0705**	**−0.0200**	**−0.0093**
Skewness	MC(10)	−0.2932	−0.1673	−0.0959
	MC(100)	−0.0544	−0.0884	−0.1192
	QMC	−0.1259	−0.0954	−0.0829
	MSE	**−0.0024**	**−0.0051**	**−0.0031**
Kurtosis	MC(10)	−0.9306	−0.7944	−0.6527
	MC(100)	−0.9915	−0.7274	−0.3822
	QMC	−0.9529	−0.7366	−0.6273
	MSE	**−0.3395**	**−0.1294**	**−0.0702**

The best performance within each statistic per sample size is highlighted in bold.

**Table 7:** The minimum L2.pdf and the corresponding bandwidth *h* of the kernel density estimation for No.IX distribution.

Method	*n*	*h*	min L2.pdf
MC	10	0.50	0.2108
QMC	10	0.30	0.0583
MSE	10	0.24	**0.0306**
MC	20	0.60	0.1300
QMC	20	0.23	0.0418
MSE	20	0.15	**0.0127**
MC	30	0.48	0.1471
QMC	30	0.21	0.0341
MSE	30	0.13	**0.0098**

The best performance within each sample size is highlighted in bold.

**Table 8:** The minimum L2.pdf and the corresponding bandwidth *h* of the kernel density estimation for No.X distribution.

Method	*n*	*h*	min L2.pdf
MC	10	0.90	0.2067
QMC	10	0.36	0.0563
MSE	10	0.29	**0.0305**
MC	20	0.46	0.1504
QMC	20	0.28	0.0379
MSE	20	0.18	**0.0119**
MC	30	0.53	0.1072
QMC	30	0.24	0.0295
MSE	30	0.15	**0.0094**

The best performance within each sample size is highlighted in bold.

**Table 9:** The minimum L2.pdf and the corresponding bandwidth *h* of the kernel density estimation for No.XI distribution.

Method	*n*	*h*	min L2.pdf
MC	10	0.71	0.1607
QMC	10	0.38	0.0628
MSE	10	0.35	**0.0441**
MC	20	0.57	0.1592
QMC	20	0.29	0.0481
MSE	20	0.22	**0.0212**
MC	30	0.76	0.1392
QMC	30	0.25	0.0382
MSE	30	0.17	**0.0137**

The best performance within each sample size is highlighted in bold.

**Table 10:** Parameter estimates of the GAST model based on $O_{3}$ data.

Parameters	$\hat{\alpha }$	$\hat{s}$	$\hat{\nu }$	$\hat{\mu }$	$\hat{\sigma }$
QMC-MLE	−0.1518	−0.2030	16.9607	1219.228	385.0162

**Table 11:** The estimation bias of the four statistics for the fitted GAST model is based on three types of RPs with size 30.

Method	Mean	Variance	Skewness	Kurtosis
MC	39.6251	−46,385.8384	−0.1111	−1.3819
QMC	−0.1250	−10,554.1427	−0.0121	−0.7845
MSE	0.0048	−579.8522	0.0005	−0.0641

**Table 12:** Parameter estimates of the GAST model based on Faithful Geyser data.

Parameters	$\hat{\alpha }$	$\hat{s}$	$\hat{\nu }$	$\hat{\mu }$	$\hat{\sigma }$
Plain-MLE	−2.4016	−0.2322	100	70.6301	8.7872

**Table 13:** The estimation biases of the four statistics for the fitted GAST model based on three types of RPs with size 50.

Method	Mean	Variance	Skewness	Kurtosis
MC	2.8239	−11.0749	0.02917	0.5070
QMC	0.0010	−2.5761	0.0010	−0.0907
MSE	0.0000	−0.1364	−0.0001	−0.0051

## Data Availability

A part of the dataset utilized for case studies in this paper is openly available in UCI Machine Learning Repository at https://archive.ics.uci.edu/dataset/360/air+quality (accessed on 15 September 2024). Another data set for analysis in this paper is obtained from R package *datasets*, named *faithful: Old Faithful Geyser Data*.
